# Commuter’s personal exposure to air pollutants after the implementation of a cable car for public transport: Results of the natural experiment TrUST

**DOI:** 10.1016/j.scitotenv.2022.160880

**Published:** 2022-12-11

**Authors:** Ricardo Morales-Betancourt, Maria A. Wilches-Mogollon, Olga L. Sarmiento, Daniela Mendez Molano, Daniela Angulo, Paola Filigrana, Julian Arellana, Luis A. Guzman, Gabriela Garzon, Nelson Gouveia, Paul Levy, Ana V. Diez-Roux

**Affiliations:** a Department of Civil and Environmental Engineering, School of Engineering, Universidad de Los Andes, Cra 1 18^a^-12, Bogotá, Colombia; b Department of Industrial Engineering, School of Engineering, Universidad de Los Andes, Cra 1 18^a^-12, Bogotá, Colombia; c School of Medicine, Universidad de Los Andes, Cra 1 18^a^-12, Bogotá, Colombia; d Universidad Manuela Beltrán, Unidad de Ingenieria Ambiental, Cra. 1 #No. 60-00, Bogotá, Colombia; e Department of Civil and Environmental Engineering, College of Engineering, Universidad del Norte, Barranquilla, Colombia; f Department of Preventive Medicine, University of Sao Paulo Medical School, Sao Paulo, Brazil; g Urban Health Collaborative, Dornsife School of Public Health, Drexel University, Philadelphia, PA, United States; h Dornsife School of Public Health, Drexel University, Philadelphia, PA, United States

**Keywords:** Personal exposure, Air pollution, Transport, Black carbon, Clean transport

## Abstract

Commuters in urban settlements are frequently exposed to high concentrations of air pollutants due to their proximity to mobile sources, making exposure to traffic-related air pollutants an important public health issue. Recent trends in urban transport towards zero- and low-tailpipe emission alternatives will likely result in decreased exposure to air pol-lutants. The TrUST (Urban transformations and health) study offers a unique opportunity to understand the impacts of a new cable car (TransMiCable) in underserved communities within uogotá, Colombia. The aims of this study are to assess the personal exposure to fine particulate matter (PM_2.5_), equivalent Black Carbon (eBC), and Carbon Monoxide (CO) in transport micro-environments and to estimate the inhaled dose per trip during mandatory multimodal trips before and after the implementation of the TransMiCable. We collected personal exposure data for Bus-Rapid-Transit (BRT) feeder buses, regular buses, informal transport, pedestrians, and TransMiCable. TransMiCable showed lower exposure concentration compared to BRT feeder and regular buses (PM_2.5_: 23.6 vs. 87.0 μg m^−3^ (*P* ≤ 0.001) and eBC: 5.2 vs. 28.2 μg m^−3^ (*P* ≤ 0.001), respectively). The mean concentration of PM_2.5_ and eBC inside the TransMiCable cabins were 62 % and 82 % lower than the mean concentrations in buses. Furthermore, using a Monte Carlo simulation model, we found that including the TransMiCable as a feeder is related to a 54.4 μg/trip reduction in PM_2.5_ inhaled dose and 35.8 µg/trip in eBC per trip. Those changes represent a 27% and 34% reduction in an inhaled dose per trip, respecti vely. Our results show that PM_2.5_, eBC, and CO inhaled dose for TransMiCable users is reduced due to lower exposure concentration inside its cabins and shorter tra vel time. The implementation of a cable car in Bogotá is likely to reduce air pollution exposure in transport micro-environments used by vulnerable populations livin g in semi informal settlements.

## Graphical abstract

**Graphical abstract:**
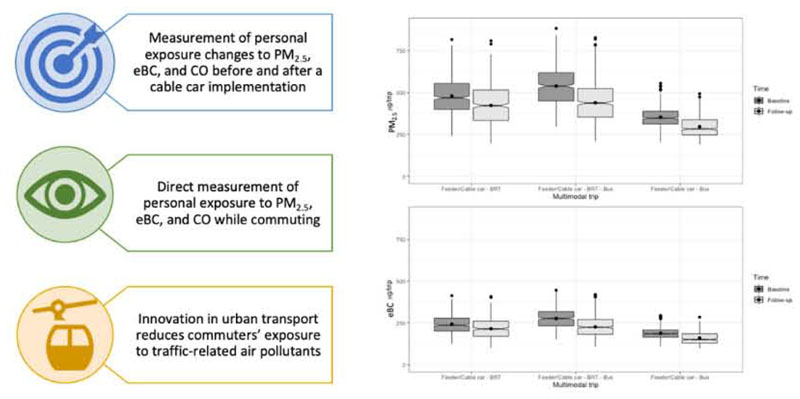

## 1 Introduction

Motorized transport is a major contributor to air pollution worldwide (Health Effects Institute, 2010). Despite several policies designed to reduce tailpipe vehicle emissions, exposure to traffic-related air pollutants such as nitrogen dioxide (NO_2_), fine particulate matter (PM_2.5_), carbon monoxide (CO), and black carbon (BC) remains a significant public health issue due to the impact of these pollutants on mortality, adverse cardiovascular and respiratory endpoints, stroke, and cancer (Health Effects Institute, 2010; Grahame and Schlesinger, 2010; Gan et al., 2011; Chen et al., 2013; Peretz et al., 2008; Maynard et al., 2007; Chen et al., 2012; Global Road Safety Facility et al., 2014). The burden of these outcomes attributed to air pollution from traffic emissions has been estimated at 184,000 deaths per year globally (Global Road Safety Facility et al., 2014). Furthermore, in I atin American cities, PM_2.5_ exposures remain a major health risk implying that urban planning and transport policies that significantly impact ambient levels could have important health implications (Gouveia et al., 2021; Targino et al., 2020).

Transport microenvironments contribute to a significant fraction of daily exposure to traffic-related air pollutants (Dons et al., 2012). In fact, daily exposure peaks occur while commuting (Gulliver and Briggs, 2004; Kaur et al., 2007). Although <10 % of daily time is spent on transport (Dons et al., 2012; Matz et al., 2018), it significantly contributes to both daily personal exposure and inhaled dose of traffic-related air pollutants (de Nazelle et al., 2012; Morales Betancourt et al., 2017; Morales Betancourt et al., 2019; Cepeda et al., 2017). For instance, a study in Belgium showed that daily commuting contributes to 21 % of personal exposure to BC and 30 % of the inhaled dose of BC, although only 6 % of the time was spent commuting (Dons et al., 2012). In cities facing air pollution problems, such as Bogotá, Colombia, a round-way trip in the Bus Rapid Transit (BRT) system accounted for nearly 60 % and 79 % of the PM_2.5_ and eBC (mass concentration of black carbon in an optical absorption method) daily dose in 2017, respectively (Morales Betancourt et al., 2017).

Several studies have also shown that motorized commuters are exposed to greater traffic-related air pollutants than users of active transport modes (Gulliver and Briggs, 2004; de Nazelle et al., 2012; Dennekcamp et al., 2002; Levy et al., 2002). For example, in a recent study in Bogota, high concentrations of PM_2.5_, eBC, and sub-micron particles (Np) were observed for all transport modes, yet the highest concentrations were observed on public transport buses (Morales Betancourt et al., 2017). Despite high levels of exposure in motorized transport, pedestrians and cyclists have higher potential inhaled doses than commuters using motorized vehicles, with cyclists experiencing the highest uptake of pollutants (Cepeda et al., 2017). In addition, a systematic review found that commuters using motorized transport lost up to one year of life expectancy over cyclists due to the combined effects of PM_2.5_ exposure and less physical activity (Cepeda et al., 2017).

Innovations in urban transport can be a means to overcome the significant environmental and health burdens associated with urban mobility. Latin America has been a center of innovation in urban transport and mobility (Sarmiento et al., 2017). For instance, 67 % of the 18 cities with aerial cable cars used as a public transport alternative worldwide are in Latin America. Γhese cable cars are used to overcome accessibility and connectivity problems, usually providing accessibility for the population living in informal settlements. In Latin America, cities such as Medellin (Colombia), La Paz (Bolivia), Mexico City (Mexico), Santiago (Chile), and Bogotá (Colombia) use aerial cable cars as part of their mass transit system (Canon Rubiano et al., 2020). One of the newest public transport alternatives in Bogotá is TransMiCable, an aerial cable car designed as an alternative for inclusive urban development to improve mobility in a socioeconomically deprived area of the city (Sarmiento et al., 2020), where travel time reductions, comfort improvements, and in-vehicle security were the benefits most valued by the 7.5 million users per year (Guzman et al., 2022).

The natural experiment TrUST (Urban transformations and health) conducted as part of the SALURBAL project (Salud Urbana en America Latina) offers a unique opportunity to understand the impact of transport systems and urban interventions on air pollution exposure through the analysis of the aerial cable car system operation in Bogotá (Sarmiento et al., 2020). The aims of this study are 1) to assess the personal exposure to air pollutants (PM_2.5_, eBC, CO) in different transport micro-environments (BRT feeder buses, regular buses, informal transport, pedestrians, and Trans-4iCable) before and after the implementation of TransMiCable project and 2) to estimate the inhaled dose per trip during multimodal mandatory trips before and after the implementation of TransMiCable. This study improves understanding of the potential impact of urban and transport interventions among vulnerable populations on microenvironment exposure by providing direct exposure measurements. This study also provides evidence relevant to policies, including those needed to achieve the United Nations’ sustainable development goal of environmental sustainability in underserved communities (ONU, 2017).

## 2 Methods

We collect personal exposure data to assess personal exposure to air pollutants (PM_2.5_, eBC, CO) in different transport micro-environments and use the estimated data and secondary data sources to estimate the mean inhaled dose per micro-environment. We then combined this information with survey data on travel behavior and used simulation modeling to derive estimates of exposures per multimodal trip.

### 2.1 Study area

The TrUST quasi-experimental natural experiment was conducted in Bogotá, the capital of Colombia, with a population of 7.4 million inhabitants in 2018 (Sarmiento et al., 2020). Bogotá, like many other cities in Latin America, faces challenges related to air pollution, as well as high-traffic congestion and long commute times (Guzman et al., 2020). Particulate matter concentrations are the most concerning air quality problem in the city as they often exceed recommended limits. During 2019, all the 13 air quality monitoring stations in Bogotá exceeded the World Health Organization (WHO) annual mean concentration guidelines for PM_10_ (15 μg/m^3^) and PM_2.5_ (5 μg/m^3^). At least four stations in the city had annual mean PM_10_ concentrations higher than 40 μg/m^3^, and two of those stations reported annual mean PM_2.5_ concentrations over 25 μg/m^3^.

Bogotá’s integrated public transport system comprises several subsystems: the BRT services, the BRT feeder bus services, the regular bus services, and TransMicable. The BRT component, TransMilenio, uses exclusive lanes for high-capacity buses (of either 140 or 250 passengers) and dedicated stations. The regular and BRT feeder buses operate on mixed traffic lanes and use roadside bus stops (Rodríguez-Valencia et al., 2019). Along with the public transport system, some areas of the city count with informal transport services, which are not part of the city public transport system and are operated by private citizens parallel to official public transport alternatives (Heinrichs et al., 2017).

**Fig. 1:**
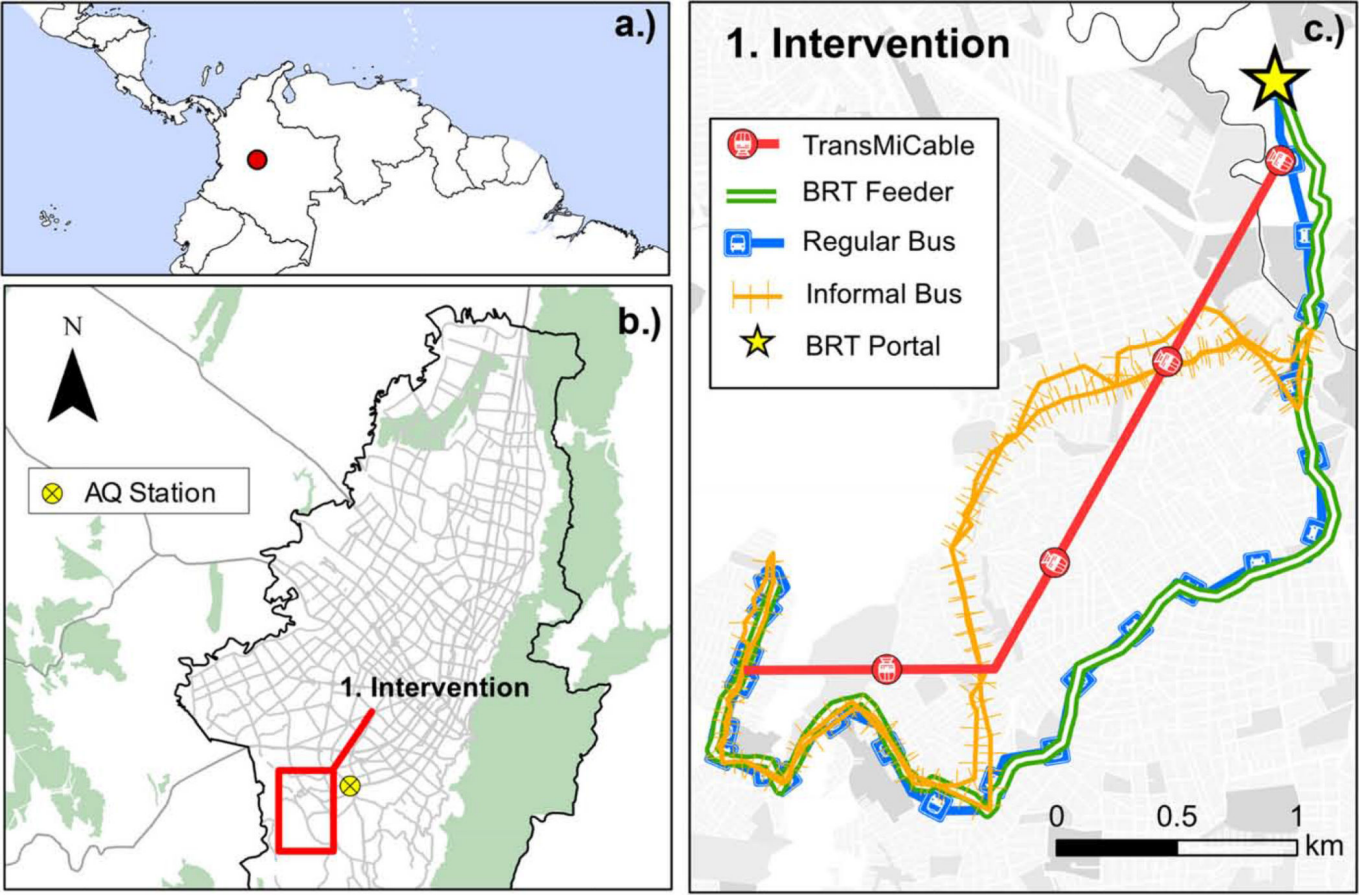
Geographic location of the study area. a.) Relative position of Bogotá within Colombia and the northern part of South America, d.) Location of the Intervention (frame denoted “1” in the inset) within the city of Bogotá limits, and the location of the closest fixed aii quality monitoring site, c.) Detailed routes for buses, informal transport, and the aerial cable car *TransMiCable* are shown for the Ciudad Bolivar intervention zone.

The natural experiment TrUST study was designed to assess the effect of implementing an aerial cable car system (TransMiCable) in the *Ciudad Bolivar* district, a low-income area in the south of the city (Fig. 1a, b). This transport system is often used in cities with geographic barriers and often serves informal settlements with high slopes (Sarmiento et al., 2020). *Ciudad Bolivar* is an administrative area of Bogotá with 616,000 inhabitants that has underg one the effects of accelerated urbanization that Bogotá has experienced since 1940. This locality is characterized by self-built settlements on highly sloped terrains on the city’s outskirts (Guzman et al., 2017). The settlements of *Ciudad B lívar* are also characterized by precarious planning, poverty, violence, and high population density. The citizens in this area face severe economic, employment, health, and mobility challenges (Sarmiento et al., 2020).

### 2.2 Intervention

TransMiCable is an electric aerial cable car transport system with one service line covering 3.34 km in length and four stations. It connects the southernmost part of *Ciudad Bolivar* to one of the end-of-line BRT stations (BRT Portal). From the BRT terminal station to the last TransMiCable station, there is an elevation gain of 270 m due to the slopes in the area. TransMiCable was inaugurated in December 2018 and mobilized 22,000 passengers per day in its first year of operation. TransMiCable is one component of an inclusive urban development program aimed at reducing socioeconomic inequalities by providing facilities and programs (e.g., parks, community centers, local market facilities, tourism office, civic services office, a housing physical improvement program, pavement of streets, and a project to reduce geomorphological risk). This area of *Ciudad Bolivar* comprises households located in neighborhoods within an 800-m airline buffer around each of the current TransMiCable stations.

At the time of the study, both BRT feeder and regular buses were diesel-powered with a capacity of up to 80 passengers. The BRT feeder buses cover routes with endpoints at the closest BRT system terminal station, while regular buses are part of the city’s public transport system. In addition, informal transport consists of passenger vehicles that are often more than two decades old and refurbished to operate with compressed natural gas.

### 2.3 Data collection

We collected information regarding exposure levels and accelerometry needed to estimate exposure levels and inhaled doses in different transport modes. For assessing personal exposure and estimating the mean inhaled dose per micro-environment, we collected in-situ PM_2.5_, eBC, and CO concentration data using portable devices in six transport microenvironments: (1) BRT buses, (2) BRT feeder buses, (3) regular buses, (4) informal transport, (5) pedestrians, and (6) TransMiCable. We also measured concomitant objective physical activity with accelerometry and global positioning system (GPS) sensors. We then used a Monte Carlo simulation model to estimate the mean inhaled dose per multimodal trip. For the simulation, we used population travel behavior information derived from the household survey of the TrUST study and the Mobility Household Survey from 2019 (Secretaria Distrital de Movilidad, 2019) and calculated the estimated mean inhaled dose per micro-environment as parameters.

The baseline measurements of exposure to air pollution in different transport micro-environments were conducted before the implementation of TransMiCable, between August and October 2018. The follow-up measurements were collected one year after the inauguration of TransMiCable, between August and November of 2019. We assessed the exposure to air pollution through visits designed to mimic the usual travel patterns of adults in the studied area. For the measurements, we carried portable air pollution monitoring devices during the data collection work.

We measured air pollution on-board of the informal vehicles, BRT feeders, regular buses, inside the TransMiCable cabins, and at the roadside waiting area of the stations of the BRT system as part of the pedestrian walking trips. The specific routes of each transport mode considered are shown in (Fig. 1c). The unit of analysis corresponds to each micro-environment of transport. One trip in each direction was performed on each sampling in the transport micro-environments. On each sampling day, we gathered information during a 2 to 3-hour sampling increments period, typically completing two trips per sampling day. Data were collected during rush and valley hours. We sampled air pollutants while traveling in transport micro-environments on 99 trips during the baseline period and on 112 trips after the implementation of TransMiCable. We carried out trip measurements on 32 BRT feeder buses, 16 in regular buses, 11 in informal transport vehicles, and 40 pedestrian walking trips during the baseline period. During the follow-up, we carried out trip measurements on 25 BRT feeder buses, 12 regular buses, ten informal transport vehicles, and 49 pedestrian walking trips on the same routes as those during the baseline period. Additionally, we carried out 16 trip measurements inside the cabins once the cable car was available.

To measure PM_2.5_ personal exposure, we used a photometric particulate matter sensor (DustTrak models 8520 and 8530, TSI Inc. MN, USA) which determines PM_2.5_ continuously, with a logging time of 1 s and a 1.0 μg/m^3^ limit of detection. The zero-reading was checked and corrected by sampling through a HEPA filter in each sampling session. The sampling flow rate was measured several times through the campaign to ensure a precise cut-off diameter. Device-specific correction factors developed in previous studies were applied to the raw PM_2.5_ DustTrak reading. These factors were 0.63 and 0.58 respectively. In addition, two gravimetric PM_2.5_ samplers Personal Environmental Monitors (PEM) (SKC Inc. PA, USA) were used in some selected sampling days to have an independent method to determine PM_2.5_The PEM pump was set at 4 LPM to achieve the required flow for a 2.5 μm cut diameter through the impaction stage. However, for several measurement days, the filter-samplers did not collect enough particle mass to determine PM_2.5_ concentration accurately. For the valid samples, the corrected DustTrak PM_2.5_ readings showed a slight +13.7 % mean bias relative to the PEM. We used portable Aethalometers (AE5l, MicroAeth, CA, USA) to measure personal exposure to eBC. This device infers eBC concentration from measurements of the rate of change in absorption of infrared light due to the continuous collection of aerosol deposits on a filter strip. The instrument was operated at a flow of 150 ml/min with data logged at 10-second intervals. The AE5l has approximately a ±0.1 μg BC/m^3^ precision at the selected flow rate. Finally, we used an electrochemical cell sensor to measure personal exposure to CO (DeltaOhm, P37AB1347 SICRAM probe) with a 1 ppm limit of detection.

We carried these devices in a backpack and traveled in the selected transport modes. The sampling inlets for these devices were located in the individuals’ breathing zone. The time resolution of the direct-reading instruments was set to 10 s. To account for variations in ambient air pollution levels during the sampling period, we used hourly data from the nearest air-quality monitoring site for the specific dates and times of the micro-environmental samplings. The air quality monitoring site location relative to the study area is shown in Fig. 1b. The devices and methods used in this study follow the methodologies proposed in previous studies (Morales Betancourt et al., 2017; Morales Betancourt et al., 2019) and are only briefly described here.

We also used Accelerometers (Actigraph GT3X+, Ft. Walton, FL.) placed on the hip to measure physical activity levels simultaneously with air pollutant concentration data. The GT3X + measures acceleration in three axes at a frequency of 30 Hz with a dynamic range of ± 8G. The accel-eration event counts have been shown to correlate well with Metabolic Equivalence Units (METs) (Freedson et al., 1998) and ventilation rate (Kawahara et al., 2011). All measured variables were synchronized at a 10 s time base. Geolocation, elevation, and speed were tracked with a GPS and recorded every second with an accuracy of ± 5 m. We used a detailed field registry to keep a precise record of events during each trip. The registered data included the moment of boarding or deboarding a given transport mode, the precise timing of walking segments, and specific situations such as passing a high-emitting vehicle and other noteworthy situations.

#### 2.3.1 Analysis of personal exposure to air pollutants

The air pollution exposure data was analyzed together with the GPS and the field registry, such that the concentration data could be analyzed in the context of the timing of events and the spatial location of the measurements. The concentration-time series from the air pollution sampling devices were synchronized using a lag-correlation analysis to ensure that all instrument signals were exactly concurrent. The BC data was corrected to account for a known loading effect caused by the accumulation of optically absorbing particles on the MicroAeth filter. As filter loading increases, the MicroAeth sensitivity reduces. To correct for this effect, we used Virkkula’s loading correction method (Virkkula et al., 2007):

(1) $$BC_{c}=BC_{nC}\times (1+k\;ATN)$$

where *BC_c_* is the corrected carbon concentration, *BC_nc_* is the uncorrected concentration, ATN is the filter attenuation, and k is the loading correction factor. This correction factor is obtained after performing simultaneous measurements with two AE5l devices operating at different flow rates. Both devices are assumed to lose sensitivity, and a least-squares method is applied to the data with Eq. (1). The optimal k found for our data is 0.005.

#### 2.3.2 Inhaled dose per unit time for each transport micro-environment

We measure the inhaled dose per minute of each air pollutant *j* ∈ *P*, where *P* is the set of air pollutants (PM_2.5_, eBC, and CO), and for each microenvironment *i* ∈ *M*, where *M* is the set of microenvironments (BRT, BRT feeder bus, regular bus, informal transport, pedestrian, TransMiCable). For the estimation, we used the measured personal exposure concentration data for each transport microenvironment, $C_{i}^{j}[{\text{μg}\;\text{m}}^{-3}],$ and the estimated inhalation rate *IR_i_* [m^3^ min^−1^] associated with the activity level detected with the accelerometers. The real respiratory track deposition of particles is likely smaller than our estimated potential inhaled dose, requiring a different experimental setup (Madueño et al., 2019). According to these definitions, the estimated inhaled dose per unit time of pollutant *j* ∈ *P* in the microenvironment *i* ∈ *M*, $D_{i}^{j}[\text{μg}\min^{-1}],$ is given by ${\hat{D}}_{i}^{j}=G_{i}^{j}IR_{i}.$ Given that estimation of inhaled dose per trip (see below) required consideration of trips on the BRT and BRT exposures were not directly measured in this study, we derived ${\hat{D}}_{BRT}^{j},\forall j\in P$ using personal exposure concentration to PM_2.5_, eBC, and CO, as well as *IR* data measured for the BRT system in another study (Morales Betancourt et al., 2019). Although the BRT exposure data is for 2017, the system buses remained unchanged until 2020 when a fleet renovation process took place, substantially reducing air pollutant concentrations inside the BRT buses and stations (Morales Betancourt et al., 2022).

We applied the Wilcoxon-Mann-Whitney nonparametric test to assess differences in mean concentration across modes, as the data collected has not a normal distribution according to the Kolmogorov-Smirnov normality test. In addition, we assessed the median difference between baseline and follow-up for a given transport mode. We conducted the analysis in *R* software version 4.1.2 (R Core Team, 2019).

### 2.4 Monte Carlo simulation model to estimate the inhaled dose per mandatory multimodal trip

We developed a Monte Carlo simulation model that generates synthetic travel times to estimate the mean inhaled dose per mandatory multimodal trip, incorporating population travel behavior information collected through the household survey of the TrUST study and the Travel Household Survey from 2019 (Freedson et al., 1998) and calculating the estimated mean inhaled dose per micro-environment as parameters.

Mandatory trips are those regular trips from (to) work or study. A multimodal trip usually combines different modes as trip stages (e.g., first, walk; then, bus ride). We simulated trips using the household TrUST survey conducted before and after the implementation of TransMiCable. Our target population comprised the inhabitants of the surrounding neighborhoods of TransMiCable stations that use one or more of the assessed micro-environments as modes of their trips (19,836 trips at baseline and 31,730 at follow-up).

#### 2.4.1 Monte Carlo simulation model parameters

We simulate the inhaled dose $D_{k}^{j}$ for each air pollutant *j* ∈ *P* per mandatory trip *k* ∈ *T*, where *T* is the set of multimodal trip types assessed and *P* is the set of air pollutants evaluated. As each trip is a conjunction of two or more of the assessed transport modes in the set (of modes) *M*, the inhaled dose is based on the measured inhaled dose of pollutant *j* in mode *i* ∈ *M* per unit time in each mode, ${\hat{D}}_{i}^{j},$ (described in Section 2.3.2), and the characteristics of mandatory trips, such as the sequence of modes *M_k_* used in a given trip *k* ∈ *T* and the travel time per mode Δ*t_i_*. The inhaled dose per trip is calculated as the sum of the contribution from the different modes in the trip, i.e.,

(2) $$D_{k}^{j}=\sum_{i\in M}{\hat{D}}_{i}^{j}\cdot \Delta t_{i},\forall k\in T,j\in P$$

where the sum runs over the multiple modes of the trip, *M_k_*, and Δ*t_i_* is the time spent at each mode. Therefore, to estimate $D_{k}^{j}$ in a way that is representative of the typical trips carried out in the area of study, it is necessary to use available data on the travel time per mode of each trip. Therefore, we used data from the TrUST Survey (Sarmiento et al., 2020) and the Mobility Household Survey for 2019 (Freedson et al., 1998) to calculate travel-time distributions for each mode of each multimodal trip.

The Mobility Household Survey is administered by Bogotá’s Mobility Secretariat and collects data on total travel times and daily trips for the entire city. From this survey, we could establish the main multimodal trips of interest for the study, the number of trips per day per multimodal trip, and the total travel time for a representative sample of our study area. Further details on the selection process of multimodal trips can be found in Appendix A. For this study, we only included two- and three-stage trips, where each stage represents a specific transport mode.

Although the Mobility Household Survey provides valuable information, it is impossible to retrieve travel time per trip-stage, only the total travel time. To circumvent this limitation, we used data from the TrUST Survey, which has detailed information for trips in the area of interest, including travel times per mode of transport and by trip. However, the sample size for the TrUST survey is much smaller than that of the Travel Household Survey. Hence, we harmonized the data from the two surveys by estimating time distributions for each transport mode for each trip from the TrUST Survey for consistency with the total travel time reported in the Travel Household Survey.

Using a kernel density estimation, we estimated the probability density function of the travel time per multimodal trip stage. Appendix B describes the methodology used to estimate travel times. We calculated the percentage of commuters at baseline and follow-up with the Travel Household Survey and the TrUST Survey.

We compared the total travel times for multimodal trips estimated with the simulation model with the total travel times from the Travel Household Survey data using the nonparametric Wilcox-Mann-Whitney to check the consistency of the travel time estimations.

#### 2.4.2 Monte Carlo simulation model

**Fig. 2:**
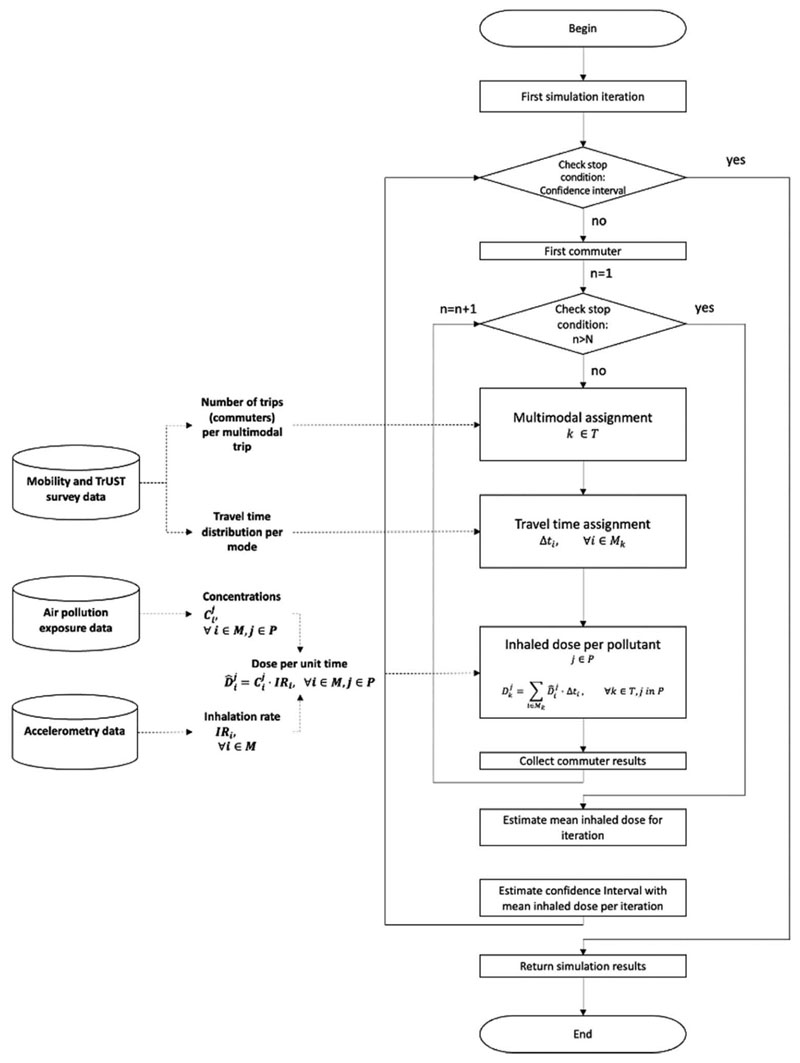
Monte Carlo simulation model flow diagram.

The model comprises several steps. First, we estimate the number of trips per day per multimodal trip and the travel time distributions for each mode of each trip. Second, the model generates the ‘first replication, representing a typical day. The model creates N commuters as trips per day for each replication and assigns them a multimodal trip, representing the trips per day estimated from the Mobility Household Survey. The commuter travels through every transport mode of the sequence, recording the travel time spent in each mode. Then, the model computes for each commuter the aggregated inhaled dose for the trip for each air pollutant. The number of commuters per trip is representative of the population under study. Third, after the last commuter finishes the trip, the model estimates the mean inhaled doses per pollutant per multimodal trip. Fourth, the model creates a new replication and estimates the mean inhaled doses for that replication. The simulation model performs several replications to guarantee a confidence interval of 95 %. After estimating the mean inhaled dose per multimodal trip for each replication, the model estimates a mean inhaled dose per multimodal trip per air pollutant with the results of all the replications and estimates confidence intervals. We designed and developed the model in the *R* software version 4.1.2 (R Core Team, 2019). Fig. 2 shows the flow diagram of the simulation model.

#### 2.4.3 Experiment design for estimating mean inhaled doses per multimodal trip

To estimate the mean inhaled dose of PM_2.5_, eBC, and C) changes, we compared the estimated inhaled dose for two-stage and three-stage multi-modal trips at baseline and follow-up. We also compared trips at baseline that contain “Bus” or “Feeder” as the first stage with those at follow-up that have “Cable car” as the first stage and maintain the other stages as in baseline. We used t-tests for each comparison. We applied the t-test to establish whether the mean trip inhaled dose differences are significant.

## 3 Results

### 3.1 Personal exposure

**Fig. 3:**
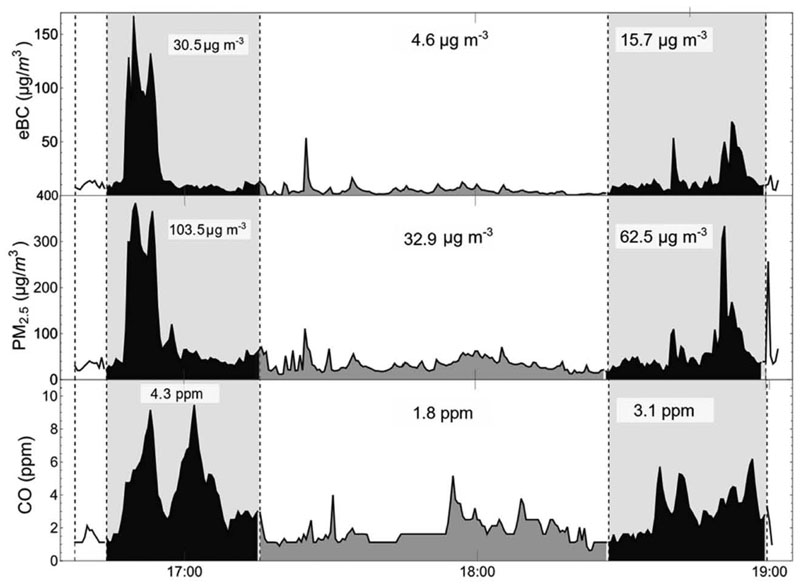
Time series of an air pollutant exposure measurement session conducted in Ciudad Bolivar on October 01, 2018. The time periods on board the BRT feeder bus are shown in black, gray shading shows time periods dining whicn the mode was ‘walking”; white corresponds to time spent at the BRT terminal station. The dotted lines represent changes in each microenvironment. The numbers in the shaded region are the mean concentration for that specific microenvironment.

**Fig. 4:**
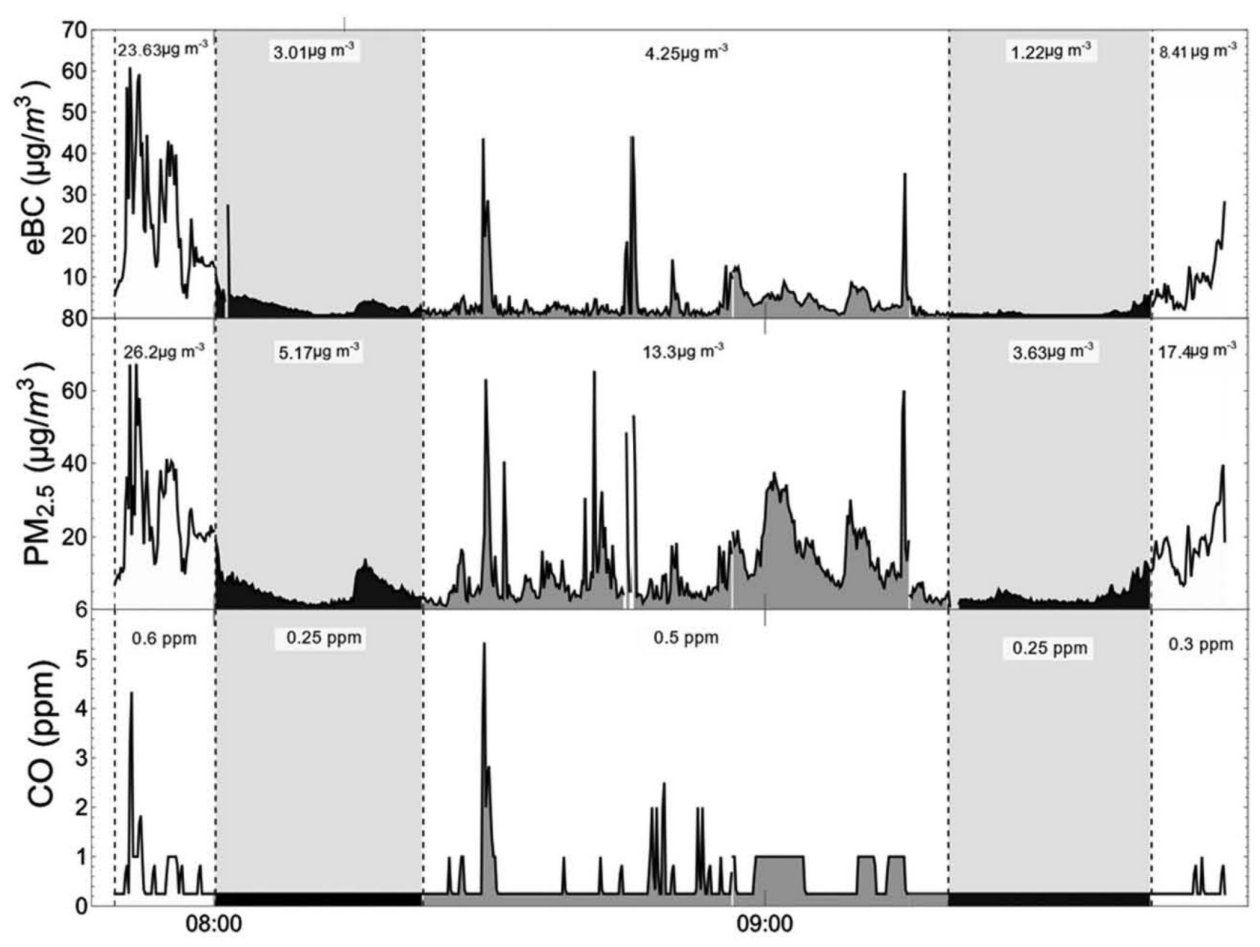
Time series of a monitoring session during follow-up conducted in Ciudad Bolivar on September 12,2019. The periods coiresponding to different stages of the trip are shown in shading. Samplings inside the TransMicable are shown in black, in gray when the people in the field were walking, and in white the position at the BRT terminal station. The dotted lines represent changes in each microenvironment. The numbers in the shaded region are the mean concentration for that specific stage in the trip.

An example post-processed data time-series obtained during sampling sessions are shown in Fig. 3 (for Feeder Bus) and in Fig. 4 (for cable-car). The data is partitioned according to the specific micro-environment being measured at the time. The concentration of PM_2.5_, eBC, and CO were often strongly correlated, suggesting a common origin for these pollutants, very likely traffic-related. Furthermore, the observations show that concentrations inside the motorized modes are often higher than outside. This can be seen in Fig. 3 as a rapid increase in the concentration of PM_2.5_, eBC, and CO when a BRT feeder bus is boarded, likely due to poor fresh air exchange rates inside the cabins of public transport buses. In contrast, while walking outdoors, exposure is often lower due to the rapid dispersion of polluted plumes, even when next to busy roads.

During the follow-up, once TransMiCable was operating, we found that the exposure to eBC, PM_2.5_, and CO measured for the TransMiCable were lower than those of any other mode considered, with a mean of 5.2 μg m^−3^ of eBC and 32.6 μg m^3^ of PM_2.5_ inside the cable car cabins. TransMiCable concentrations were significantly different from those in all the other modes, including pedestrian mode. A typical monitoring data time series when TransMiCable is sampled shows that the moments inside the cable car cabins have low levels of air pollutants and exhibit lower variations (Fig. 4). Detailed analysis of the measurements shows that increases in the in-cabin air pollutant concentrations are due to the proximity of the intermediate TransMiCable stations, where the laterally sliding doors of the cabin open up, allowing the entry of outside air.

Data obtained from the nearby air quality monitoring site shows that urban ambient PM_2.5_ concentrations only varied slightly between the baseline (19.7 μg m^−3^) and follow-up (16.7 μg m^−3^) campaigns. This suggests that the changes observed in-cabin are more likely explained by the micro-environmental conditions and were not significantly impacted by the urban ambient levels.

**Fig. 5:**
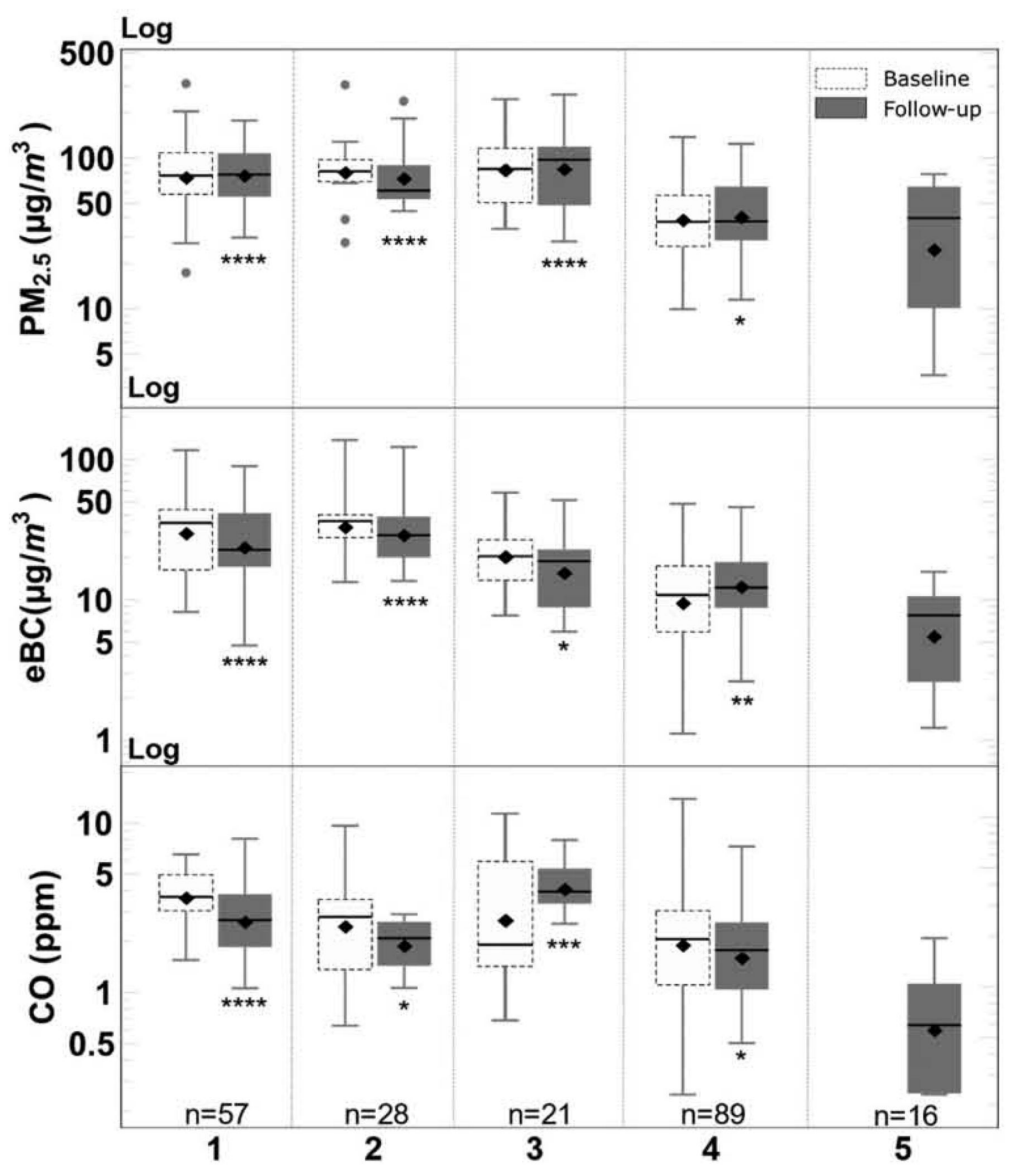
Exposure concentration for PM_2.5_ (in μg m^−3^), eBC (μg m^−3^), and Cu (in ppm) in each transport mode, (1) BRT Feeder, (2) Regular Bus (3) informal transport, (4) pedestrians, and (5) TransMiCable, for both data collection moments (baseline and follow up). The diamond marker is the mean concentration for each category; the horizontal full line represents the median. A nonparametric Mann-Whitney mean difference test was performed. The test compares TransMiCable with each of the transport modes only at follow-up. *P ≤ 0.05, **P ≤ 0.01, ***P ≤ 0.001, ***P ≤ 0.0001.

Fig. 5 synthesizes the observed concentrations for the 211 monitored microenvironments measurements. We reported high exposure concentrations in motorized modes such as BRT feeder buses, regular buses, and informal vehicles. The mean PM_2.5_ concentration inside buses was 87.0 μg m^−3^ across baseline and follow-up, and eBC 28.2 μg m^−3^ for either bus type. In both sampling campaigns, the two bus subsystems analyzed, BRT feeder and regular buses, were found to have similar PM_2.5_ concentrations, with statistically indistinguishable medians. eBC concentration across modes shows larger variability, with informal transport vehicles having the lowest eBC among motorized modes, likely due to the use of refurbished Compressed natural gas (CNG)-powered engines, which are expected to produce little sooL These concentrations are consistent with what has been observed in other studies in the city, which have reported median in-bus PM_2.5_ concentrations between 79.3 and 92.9 μg m^−3^, as well as eBC concentrations between 55 and 74 μg m^−3^ (Morales Betancourt et al., 2017).

Compared to BRT feeder buses, the mean in-cabin TransMiCable PM_2.5_ and eBC concentrations were 30 μg m^−3^ and 12 μg m^−3^ lower, respectively. On average, the measured concentrations inside the cable car cabins are slightly lower than the background concentrations.

Pedestrian exposure was the lowest among the modes available before the cable implementation during the baseline. The relatively lower exposure for pedestrians has been observed in other studies, likely due to the better ventilation when compared to the enclosed cabins of other transport modes, as shown in Fig. 5.

We observed significant statistical differences across all modes (*P* < 0.05) except for the BRT feeder and regular buses. This difference is consistent with the fact that both bus services are diesel-powered and cover similar routes. A summary of the significance of this test is included in Appendix C.

### 3.2 Simulated inhaled dose

#### 3.2.1 Selection of multimodal trips and commuters distribution

We used the methodology described in Section 2.4.1 and Appendix A to select the multimodal trips evaluated and the commuters’ distribution. Due to changes in the use of multimodal trips between baseline and follow-up and the inclusion of the cable car as a follow-up transport mode, we were able to assess the inhaled dose for five multimodal trip types at baseline and follow-up. We assessed the following multimodal trips for baseline “Feeder – Bus”, “Feeder – BRT”, “Bus - BRT”, “Informal – Bus”, and “Feeder – BRT – Bus”; and follow-up “Feeder – Bus”, “Feeder – BRT”, “Cable car – Bus”, “Cable car – BRT”, “Cable car – BRT – Walk”, “Cable car – BRT – Bus”. Table 1 shows commuters’ distribution for each trip at baseline and follow-up.

For the statistical analysis, we compared multimodal trips “Feeder – Bus”, “Feeder – BRT”, and “Feeder – BRT – Bus” at baseline with “Cable car - Bus”, “Cable car – BRT”, and “Feeder – BRT – Bus” at follow-up, respectively. In addition, multimodal trips “Bus – BRT”, “Informal – Bus”, and “Cable car – BRT – Walk” were evaluated either at baseline or followup. However, their results are used only in the two-stage and three-stage comparisons due to a lack of information.

#### 3.2.2 Travel time evaluation

Our travel time estimations fall within the confidence interval of the Travel Household Survey, demonstrating the consistency between both surveys at the total travel time estimations level. Furthermore, baseline and follow-up estimated travel times fall within the same confidence interval (CI), suggesting no significant changes in the travel time for multimodal trips when transport modes did not change because of the intervention. However, trips that include the cable car in one stage have shorter travel times than those that do not include the cable car. For the baseline campaign, the mean travel time for two-stage trips was 125.5 min (95 % CI: 120.03–131.08), while the mean travel time for three-stage trips was 137.9 min (95 % CI: 133.16–142.57). For the follow–up campaign, the mean travel time for two-stage and three-stage trips that included the cable car is 98.4 (95 % CI: 95.29–101.49). This value is 102.8 (95 % CI: 100.60–104.92) for trips that do not include the cable car as a transport mode.

With the collected information from the baseline and follow-up campaigns, we estimated the inhaled dose per trip for up to ten modal combinations. Appendix D summarizes the travel time estimations for the multimodal trips evaluated.

#### 3.2.3 Inhaled dose per trip estimation

**Fig. 6:**
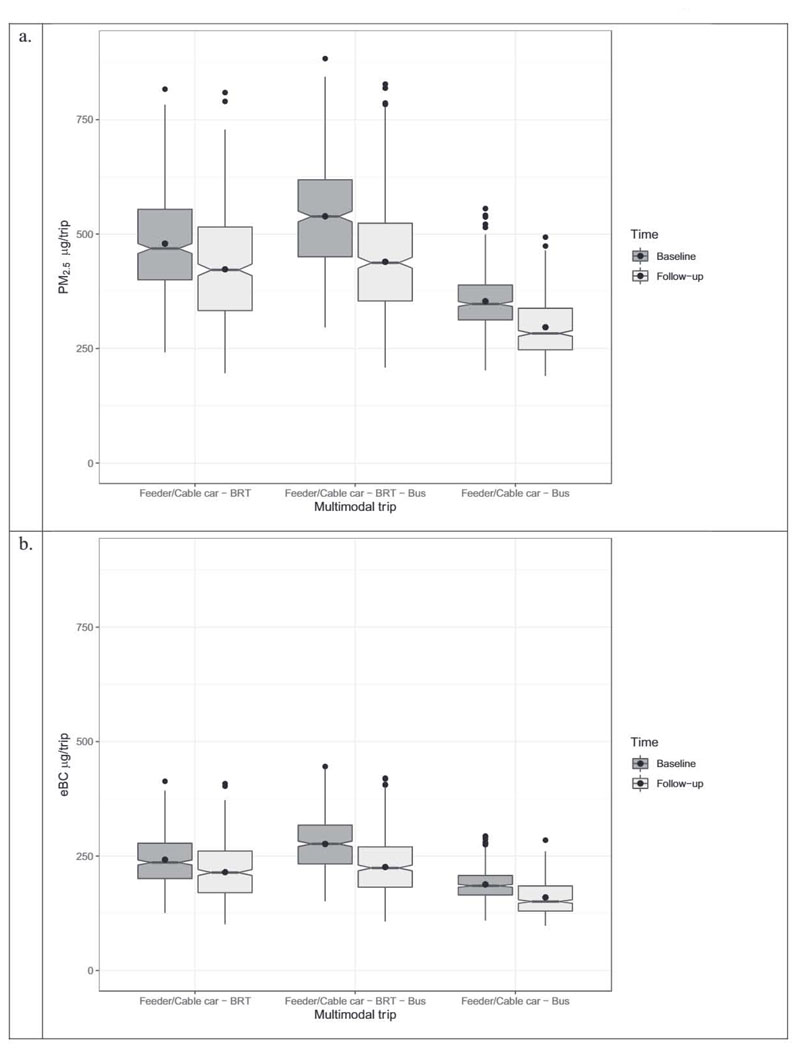
Estimated inhaled dose per multimodal trip at baseline and follow-up for a) PM_2.5_ and b) eBC. Multimodal trips considered for baseline were: Feeder-BRT, Feeder-BRT-Bus, and Feeder-Bus. Multimodal trips considered for follow-up were: Cable car-BRT, Cable car-BRT-Bus, and Cable car-Bus. The cable car is a feeder of the BRT system in the follow-up measurement.

We estimated the inhaled doses of PM_2.5_, eBC, and CO per trip for each multimodal trip per campaign. Table 2 summarizes the potential inhaled dose estimations (mean and quantiles) for each multimodal trip, as well as two-stage and three-stage trips overall. Fig. 6 summarizes the inhaled dose estimations per trip (mean and quantiles) for PM_2.5_, eBC, and CO for each two-stage multimodal trip before and after the implementation of TransMiCable.

The two-stage trip “Feeder-Bus” has the lowest inhaled dose per trip (PM_2.5_: 103.7 μg/trip; eBC: 54.3 μg/trip; CO: 4.6 mg/trip) among all baseline multimodal trips. Likewise, the comparable two-stage trip “Cable car-Bus” has the lowest inhaled dose per trip (PM_2.5_: 77.3 μg/trip; CO: 40.7 μg/trip; eBC: 3.6 mg/trip) among all follow-up and baseline multimodal trips.

When analyzing trips by the number of stages at baseline, the estimated PM_2.5_ inhaled dose in two-stage trips is significantly lower than in three-stage trips (148.4 μg/trip vs. 197.0 μg/trip). Likewise, the estimated eBC inhaled dose is significantly higher for three-stage trips than for two-stage trips (96.4 μg/trip vs. 68.4 μg/trip). When analyzing at follow-up, two-stage trips have a smaller mean inhaled dose than three-stage trips overall air pollutants assessed (PM_2.5_: 114.0 μg/trip vs. 143.6 μg/trip; eBC: 57.3 μg/trip vs. 69.1 μg/trip; CO: 4.5 mg/trip vs. 5.2 mg/trip).

Lastly, as shown in Fig. 6, trips that include “Cable car” present a significantly lower mean inhaled dose per trip in PM_2.5_ and eBC, compared with the comparable multimodal trip with “feeder” as the first stage.

## 4 Discussion

Our results show that innovations in urban transport, such as TransMiCable, can reduce commuters’ exposure to traffic-related air pollutants. Specifically, we found that the cable car is the transport mode with the lowest personal exposure to PM_2.5_ and eBC. The concentration inside the cable car cabins is even lower than for pedestrians, which is surprising as most studies have shown higher concentration inside the cabins of transport modes. The mean in-cabin cable car concentrations of PM_2.5_ and eBC were 62 % and 82 % lower than those found in buses, respectively (BRT feeder or regular buses). Furthermore, cable car users experienced concentrations of PM_2.5_ and eBC 3.1 and 4.2 times lower, respectively, when compared to informal transport users. In addition, pedestrians have the second-lowest exposure.

TransMiCable reduced commuting times, traveling 3.34 km in only 13 to 17 min. This represents a 50 % reduction in commute time compared to when traveling the same distance using other transport modes. This reduction in commuting times and the much lower exposure concentrations result in a decrease in inhaled doses for cable car users. The cable car impact on the inhaled dose was most evident when we analyzed the cable car as a BRT feeder substitute. The de-crease in inhaled dose experienced by commuters in “Feeder -Bus” multimodal trips that replaced the first bus stage by the cable car, i.e., “Cable car - Bus” combination, was 26.4 μg/trip and 13.6 μg/trip lower for PM_2.5_ and eBC, respectively. These reductions in inhaled dose are relevant considering they are driven mainly by a change of transport mode choice. The decrease in the PM_2.5_ inhaled dose is roughly 12 % of the 227 μg/trip (Morales Betancourt et al., 2019) intake dose estimated for a person exposed for 24 h to the daily PM_2.5_ WHO guideline concentrations. The implementation of TransMiCable and its urban transformations in Bogotá are likely to significantly reduce air pollution exposure for populations living in semi-informal settlements in transport microenvironments as it is related to a shift in transport modes and a reduction in waiting and travel times for the users of TransMiCable.

Recent research has also demonstrated significant reductions in personal exposure to air pollutants following a technological upgrade of the BRT fleet (Morales Betancourt et al., 2022). The commuter exposure reductions achieved with the implementation of TransMiCable further contribute to a decrease in air pollution exposure in the city and suggest that interventions to implement cleaner transport alternatives can have rapid and substantial benefits.

The results of this study are consistent with previous literature showing lower concentrations of traffic-related air pollutants for commuters using electric-powered public transport modes. In particular, Andersen et al. (2019) found that commuters on an electric train have 83-, 97-, and 53-times lower exposures to BC, ultrafine particles, and PM_2.5_ than those commuting on a diesel train. Similarly, Liu et al. (2015) found that commuters in an electric subway have 31 times lower PM_2.5_ exposure than commuters in a diesel bus. Similarly, personal exposure to PM_2.5_, ultrafine particles, and BC in a hybrid diesel/electric bus was significantly lower than in diesel buses (van Ryswyk et al., 2020). Although the aerial cable car and other electric public transport modes may differ in some features, they are similar in combustion-related air pollutants, given that they do not use fuel to power the engines.

Although the impact of the cable car on exposure to traffic-related air pollutants in transport microenvironments is limited to its riders, given that a little over twenty thousand passengers use this transport mode per day and the low exposure levels reported in our work, we would expect a significant health impact of the cable car implementation for the population in the study area. In addition, given the high demand for this transport alternative, we expect the implementation of similar projects in the city to expand the effects of reduced commuter exposure to other communities. Today, there are plans to build nine additional cable car lines in Bogotá.

Urban transformations such as the cable car have emerged as an alternative to connect underserved neighborhoods located on terrains where steep slopes and hilly topography limit the development of other forms of transport (van Ryswyk et al., 2020; Carlet, 2016). Besides the positive environmental impacts of this system shown in this study, several social impacts are also expected due to improvements in the access of the served neighborhoods to other locations, as well as increased subjective well-being and social capital, and greater access to work and education opportunities that might enhance the overall quality of life (Carlet, 2016). These results are especially important for informal settlements, where is estimated over 1 billion people live worldwide, many of them in hilly topography. Thus, the implementation of cable car systems along with further urban transport modernization initiatives in Bogotá, including the operation of a large fleet of electric-powered buses, the BRT fleet renewal process, and the implementation of other cable car systems, will likely result not only in substantial lower commuter exposures but also in positive air pollution, health, and social impacts.

## 5 Limitations

Despite the strengths of this study, there are some limitations. First, we did not estimate changes in commuters’ health outcomes associated with re-duced exposure to air pollutants due to the introduction of the cable car. While no previous studies assess the health impacts of this transport innovation, previous studies have documented health benefits resulting from reductions in traffic-related air pollution due to interventions to reduce traffic volume and congestion in London and Stockholm (Johansson et al., 2009; Tonne et al., 2008). Lastìy, the inhaled dose per trip obtained in this study might be slightly underestimated since this was only estimated for mandatory trips, usually trips for education or work purposes. However, according to the Bogotá Travel Household Survey, trips for education and work represent >48 % of daily trips in Bogotá (Secretaria Distrital de Movilidad, 2019).

## 6 Conclusion

We assessed the changes in personal exposure to traffic-related air pollutants attributable to the implementation of a new cable car system in Bogotá. The assessment was performed by combining in-situ measurements of personal exposure of PM_2.5_, eBC, and CO in several transport mode alter-natives, including the cable car. The field campaigns were carried out in two stages, one before (baseline) and one after (follow-up) the implementation of the cable car system. These measurements were then combined with detailed travel patterns information derived from two surveys, a city-wide Travel Household Survey and a smaller survey focused on the study area carried out specifically for this project

We observed that the concentration of all pollutants inside the cable car cabins was the lowest among all the transport modes considered. The average concentration of PM_2.5_ was 32.6 μg/m^3^, and for BC was 5.2 μg/m^3^. These values are much lower than those observed inside the public transport buses in the area, for which the PM_2.5_ and eBC concentrations were 87.0 μg/m^3^ and 28.2 μg/m^3^, respectively.

The estimation of the inhaled dose for commuters in the study area through a Monte Carlo simulation model shows that for trips where the cable car substituted a feeder ride, there are significant reductions in inhaled dose for all air pollutants analyzed. For example, the net reduction in inhaled dose amounts to 25.5 μg/trip for PM_2.5_ and 13 μg/ trip for eBC. Similarly, significant reductions were observed for other multi-stage trips for which a cable car ride replaced one such stage after it was implemented.

Findings from this study have important public health implications. First, cable car interventions might be part of an agenda of urban transformations in developing countries contributing to ambient, health, and social benefits.

Due to cleaner technology, the implementation of aerial cable cars might contribute to reductions in ambient air pollutants, thus contributing to the Sustainable Development Goals (SDG11) in low and middle-income countries. Second, this study shows that the cable car also results in lower commuters’ exposure to air pollution in transport microenvironments, likely resulting in potential health benefits for users. Third, social benefits for underserved populations might result from this transport intervention, especially by connecting them with greater opportunities to improve their living conditions. Fourth, the results of this study support informed decision-making to implement additional cable cars in underserved and poorly connected neighborhoods in Bogotá and similar urban areas in Colombia.

## Supplementary Material

Appendix

## Figures and Tables

**Table 1:** Distribution of commuters according to the mode combination used in multimodal trips.

Percentage of trips per multimodal trip		
Mode combination	Base.ine	Follow-up
Feeder – Bus	1.32 %	0.49 %
Feeder – BRT	84.61 %	39.74 %
Bus – BRT	6.19 %	–
informal – Bus	3.73 %	–
Feeder – 3RT – Bus	4.14 %	–
Cable car – Bus		1.47 %
Cable car BRT	–	40.32 %
Cable car BRT – Walk	–	8.66 %
Cable car – BRT – Bus	–	9.33 %
Total commuters	19,836	31,730

**Table 2:** Estimated inhaled dose per trip $\left(D_{trip}^{j}\right)$ for air pollutants considered in this study.

Inhaled dose per trip												
Type of multimodal trip	PM_2.5_ dose (μg/trip)				eBC dose (μg/trip)				CO dose (mg/trip)			
	Baseline		Follow-up		Baseline		Follow-up		Baseline		Follow-up	
	Mean	Median (Q_1_–Q_3_)	Mean	Median (Q_1_–Q_3_)	Mean	Median (Q_1_–Q_3_)	Mean	Median (Q_1_–Q_3_)	Mean	Median (Q_1_–Q_3_)	Mean	Median (Q_1_–Q_3_)
Feeder – BRT	166.1	166.7 (163–175)	153.1	lb7.C (149–158)	81.0	80.8 (79–85)	74.5	76.8 (72–77)	6.3	6.3 (6.l–6.5)	5.8	5.9 (5.7–6.))
Feeder – BRT – Bus	2Ü1	202.2 (198–203)	–	–	100.7	101.6 (100 –102)		–	8.2	8.3 (7.8–8.3)	–	–
Feeder – Bus	103.7	104.7 (1 2–105)	89.9	91.0 (88–95)	54.3	54.7 (53–55)	47.2	48.1 (46–50)	4.6	4.7 (4.6–4.7)	4.1	4.3 (4.0–4.4)
Cable car - BRT^a^	–	–	141.8	140.3 (134–144)	–	–	68.7	68.7 (65–70)	–	–	5.1	5.0 (4.8–5.1)
Cable car BRT Bus	–	–	144.2	147.4 (139–148)	–	–	71.4	72.6 (68–73)	–	–	5.2	5.3 (5.0–5.4)
Cable car – Bus	–	–	77.3	76.7 (72–79)	–	–	40.7	4J.3 (37–42)	–	–	3.6	3.5 (3.3–3.7)
Two-stage trips	148.4	144.9 (112–180)	114.0	117.3 (83–148)	68.4	65.2 (55–90)	57.3	60.1 (43–72)	6.1	6.1 (5.2–7.1)	4.5	4.7 (3.8–5.5)
Three-stage trips	197.0	189.5 (187–201)	143.6	145.7 (138–148)	96.4	93.7 (90–101)	69.1	70.1 (68–73)	7.6	7.5 (7.3–8.2)	5.2	5.3 (5.0–5.4)

Note 1: Values in blank represent insufficient data to estimate those multimodal trips’ travel time in each campaign. Note 2: We only include multimodal trips with at least two stages, as those are the ones that include motorized vehicles or the cable car.

a The cable car is a feeder of the BRT system in the follow-up measurement.

## Data Availability

Data will be made available on request.

## References

[R1] Andersen MHG, Johannesson S, Fonseca AS, Clausen PA, Saber AT, Roursgaard M (2019). Exposure to air pollution inside electric and diesel-powered passenger trains. Environ Sci Technol.

[R2] Canon Rubiano L, Portabales Gonzalez I, Flor L, Duarte D, Sierra Valdivieso L (2020). Urban Aerial Cable Cars as Mass Transit Systems: Case Studies, Technical Spedfications, and Business Models. Lima.

[R3] Carlet F (2016). An overview of aerial ropeway transit and its potential in urban environments. SBE2016 Towards Post Carbon Cities.

[R4] Cepeda M, Schoufour J, Freak-Poli R, Koolhaas CM, Dhana K, Bramer WM (2017). Levels of ambient air pollution according to mode of transport: a systematic review. Lancet Public Health.

[R5] Chen R, Samoli E, Wong CM, Huang W, Wang Z, Chen B (2012). Associations between short-term exposure to nitrogen dioxide and mortality in 17 Chinese dries: the China air pollution and health effects study (CAPES). Environ.

[R6] Chen H, Goldberg MS, Burnett RT, Jerrett M, Wheeler AJ, Jilleneuve PJ (2013). Long-term exposure to traffic-related air pollution and cardiovascular mortality. Epidemiology.

[R7] de Nazelle A, Fruin S, Westerdahl D, Martinez D, Ripoll A, Kubesch N (2012). A travel mode comparison of commuters’ exposures to air pollutants in Barcelona. Atmos Environ.

[R8] Dennekamp M, Mehenni OH, Cherrie JW, Seaton A (2002). Exposure to ultrafine particles and PM 2.5 in different micro-environments. Ann Occup Hyg.

[R9] Dons E, Int Panis L, van Poppel M, Theunis J, Wets G (2012). Personal exposure to black carbon in transport microenvironments. Atmos Environ.

[R10] Freedson PS, Melanson E, Sirard J (1998). Calibration of the computer science and applications. Inc Accelerometer Med Sci Sports Exerc.

[R11] Gan WQ, Koehoom M, Davies HW, Demers PA, Tamburic L, Brauer M (2011). Longterm exposure to traffic-related air pollution and the risk of coronary heart disease hospitalization and mortality. Environ Health Perspect.

[R12] Global Road Safety Facility, Institute for Health Metrics and Evaluation, The World Bank, Institute for Health Metrics and Evaluation (2014). Transport for Health:The Global Burden of Disease From Motorized Road Transport.

[R13] Gouveia N, Kephart JL, Dronova L, McClure L, Granados JT, Betancourt RM (2021). Ambient fine particulate matter in latin american cities: levels, population exposure, and assodated urban factors. Sci Total Environ.

[R14] Grahame TJ, Schlesinger RB (2010). Cardiovascular health and particulate vehicular emissions: a critical evaluation of the evidence. Air Qual Atmos Health.

[R15] Gulliver J, Briggs DJ (2004). Personal exposure to particulate air pollution in transport microenvironments. Atmos Environ.

[R16] Guzman LA, Oviedo D, Bocarejo JP (2017). City profile: the Bogotá metropolitan area that never was. Cities.

[R17] Guzman LA, Arellana J, Alvarez V (2020). Confronting congestion in urban areas: developing sustainable mobility plans for public and private organizations in Bogotá. Transp Res Part A Policy Prad.

[R18] Guzman LA, Cantillo-Garcia VA, Arellana J, Sarmiento OL (2022). User expectations and perceptions towards new public transport infrastructure: evaluating a cable car in Bogotá. Transportation (Amst).

[R19] Health Effects Institute (2010). Traffic-Related Air Pollution: A Critical Review of the Literature on Emissions, Exposure, and Health Effects.

[R20] Heinrichs D, Goletz M, Lenz B (2017). Negotiating territory: strategies of informal transport operators to access public space in urban Africa and Latin America. Transp Res Procedia.

[R21] Johansson C, Burman L, Forsberg B (2009). The effects of congestions tax on air quality and health. AtmEn.

[R22] Kaur S, Nieuwenhuijsen MJ, Colvile RN (2007). Fine particulate matter and carbon monoxide exposure concentrations in urban street transport microenvironments. Atmos Environ.

[R23] Kawahara J, Tanaka S, Tanaka C, Aoki Y, Yonemoto J (2011). Estimation of daily inhalation rate in preschool children using a tri-axial accelerometer a pilot study. Sci Total Environ.

[R24] Levy JI, Dumyahn T, Spengler JD (2002). Particulate matter and polycyclic aromatic hydrocarbon concentrations in indoor and outdoor microenvironments in Boston, Massachusetts. Journal of Exposure Sdence & Environmental Epidemiology.

[R25] Madueño L, Kecorius S, Löndahl J, Müller T, Pfeifer S, Haudek A (2019). A new method to measure real-world respiratory tract deposition of inhaled ambient black carbon. Environ PoΠuL.

[R26] Matz CJ, Stieb DM, Egyed M, Brion O, Johnson M (2018). Evaluation of daily time spent in transportation and traffic-influenced microenvironments by urban Canadians. Air Qual Atmos Health.

[R27] Maynard D, CouU BA, Gŗyparis A, Schwartz J (2007). Mortality risk assodated with shortterm exposure to traffic particles and sulfates. Environ Health Perspect.

[R28] Morales Betancourt R, Galvis B, Balachandran S, Ramos-Bonilla JP, Sarmiento OL, Gallo-Murcia SM (2017). Exposure to fine particulate, black carbon, and particle number concentration in transportation microenvironments. Atmos Environ.

[R29] Morales Betancourt R, Galvis B, Rincón-Riveros JM, Rincón-Caro MA, Rodríguez-Valencia A, Sarmiento OL (2019). Personal exposure to air pollutants in a bus rapid transit system: impact of fleet age and emission standard. Atmos Environ.

[R30] Morales Betancourt R, Galvis B, Mendez-Molano D, Rincón-Riveros JM, Contreras Y, Montejo TA (2022). Toward cleaner transport alternatives: reduction in exposure to air pollutants in a mass public transport. Environ Sci Technol.

[R31] ONU (2017). Nueva Agenda Urbana.

[R32] Peretz A, Sullivan JH, Leotta CA, Sands FN, Allen J, Carlsten C (2018). Diesel exhasut inhlation elicits acute vasoconstriction in vivo. Environ Health Perspect.

[R33] R Core Team (2019). R: A language and environment for statistical computing.

[R34] Rodríguez-Valencia A, Rosas-Satizabal D, Paris D (2019). Importance-Performance Analysis in Public Transportation: Methodological Revision for Practical Implementation.

[R35] Sarmiento OL, Siri J, Rodríguez D, Higuera-Mendieta DD, Gonzalez S, Montero S (2017). Sustainable Transport and Urban Health: The Lessons of Latin America. Bogotá.

[R36] Sarmiento OL, Higuera-Mendieta D, Wilches-Mogollon MA, Guzman LA, Rodríguez DA, Morales R (2020). Urban transformations and health: methods for TrUST— a natural experiment evaluating the impacts of a mass transit cable car in Bogotá, Colombia. Front Public Health.

[R37] Secretaria Distrital de Movilidad (2019). Encuesta de Movilidad 2019. Bogotá.

[R38] Targino AC, Krecl P, Cipoli YA, Oukawa GY, Monroy DA (2020). Bus commuter exposure and the impact of switching from diesel to biodiesel for routes of complex urban geometry. Environ Pollut.

[R39] te Liu W, Ma CM, Liu IJ, Han BC, Chuang HC, Chuang KJ (2015). Effects of commuting mode on air pollution exposure and cardiovascular health among young adults in Taipei, Taiwan. Int J Hyg Environ Health.

[R40] Tonne C, Beevers S, Armstrong B, Kelly F, Wilkinson P (2008). Air pollution and mortality benefits of the London congestion charge: spatial and socioeconomic inequalities. Occup Environ Med.

[R41] van Ryswyk K, Evans GJ, Kulka R, Sun L, Sabaliauskas K, Rouleau M (2020). Personal exposures to traffic-related air pollution in three Canadian bus transit systems: the urban transportation exposure study. J Expo Sci Environ Epidemiol.

[R42] Virkkula A, Mäkelä T, Hillamo R, Yli-Tuomi T, Hirsikko A, Hämeri K (2007). A simple procedure for correcting loading effects of aethalometer data. J Air Waste Manag Assoc.

