# Supplementary material for: Commuter’s personal exposure to air pollutants after the implementation of a cable car for public transport: Results of the natural experiment TrUST
Source: Sci Total Environ. Author manuscript; Available in PMC 2024 Dec 8. (PMC7616957; doi:10.1016/j.scitotenv.2022.160880)
Supplement: Appendix [file EMS160159-supplement-Appendix.pdf]

Appendix A. Flow diagram of selection of multimodal trips

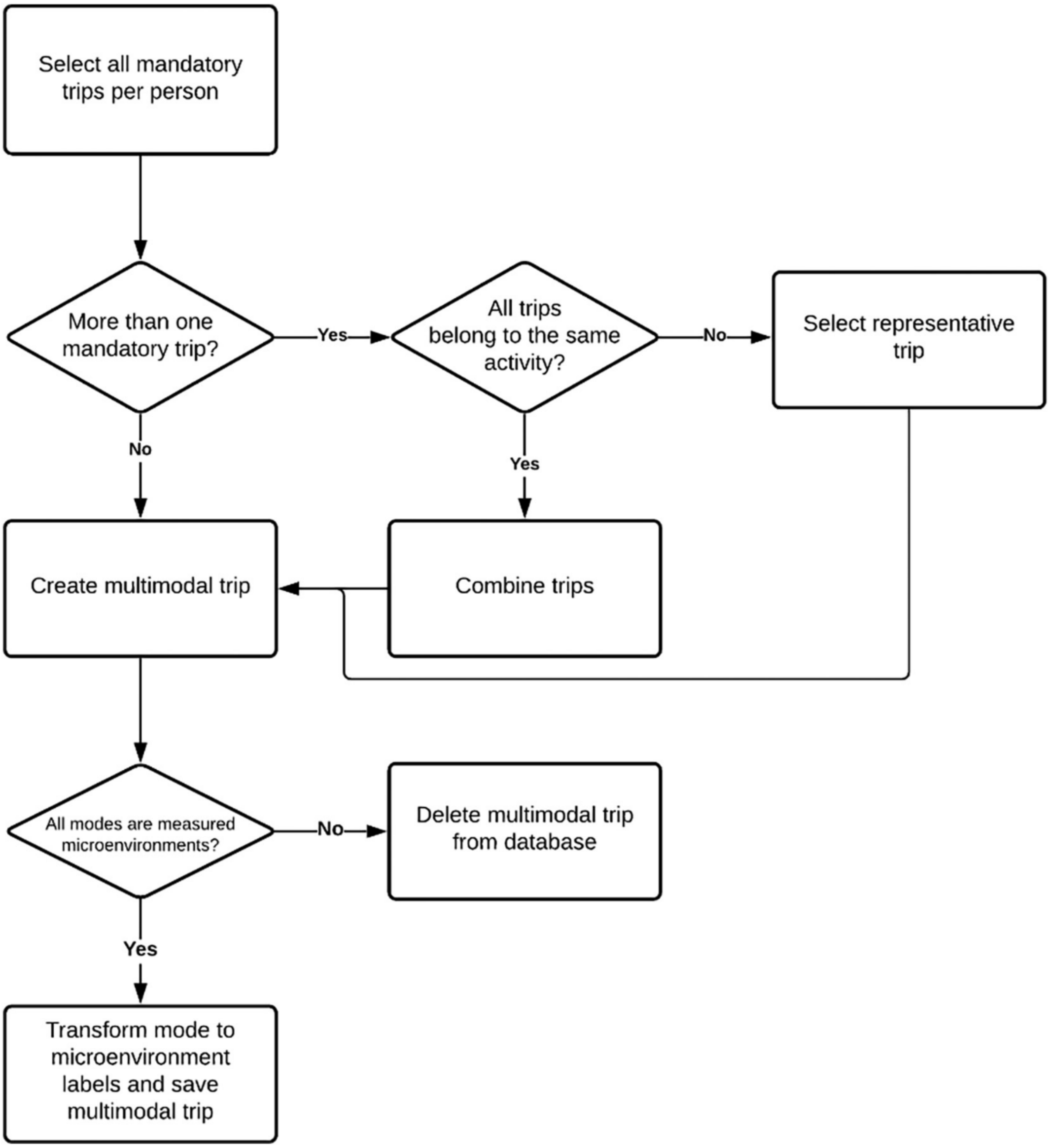

Appendix B. Flow diagram for estimation of travel times

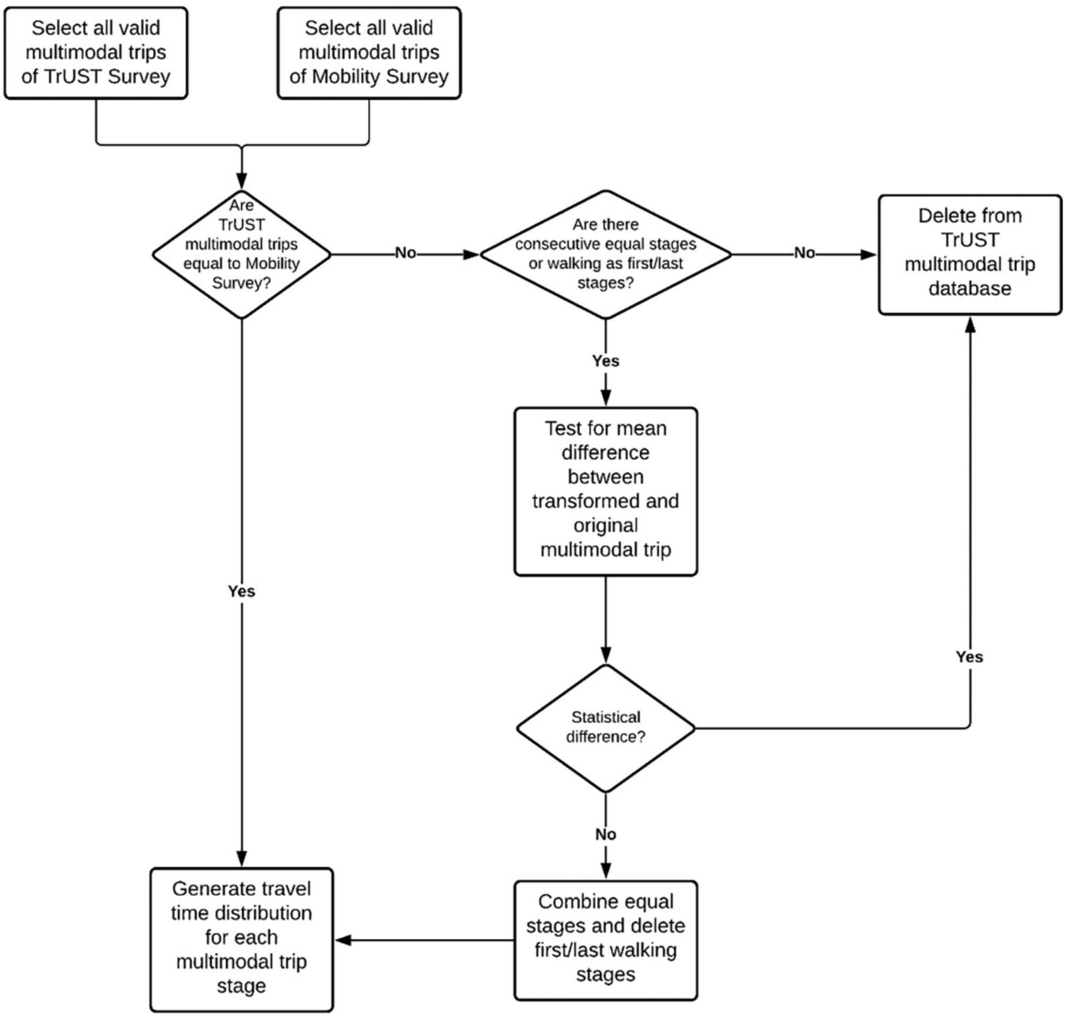

Appendix C. P values resulting from the Wilcoxon-Mann-Whitney test. Significance is observed for each of the tests between modes, considering a 95 % confidence interval

Wilcoxon-Mann-Whitney test for equality of means					
P-value	BRT feeder	Regular bus	Informal	Cable car	Pedestrian
BRT feeder	–	0.058	0.030	2.7.E − 06	1.4.E − 14
Regular bus	–	–	9.2.E − 05	1.5.E − 07	2.2.E − 15
Informal	–	–	–	2.2.E − 04	1.4.E − 03
Cable car	–	–	–	–	0.022
Pedestrian	–	–	–	–	–

Appendix D. Travel time per multimodal trip

Travel time per modal share				
Modal share	No. stages	Travel household survey	Baseline estimated	Follow up estimated
		Travel time (minutes)	Travel time (minutes)	Travel time (minutes)
		95 % CI	95 % CI	95 % CI
Feeder – Bus	2	[0–227.06]	[108.17–115.54]	[[illegible]03.33–110.[illegible]2]
Feeder – BRT	2	[98.73–111.72]	[105.98–111.63]	[[illegible]00.50–105.[illegible]5]
Bus – BRT	2	[64.32–161.68]	[114.19–154.91]	–
Informal – Bus	2	–	[108.17–115.54]	–
Feeder – BRT – Bus	3	[36.75–198.24]	[140.42–151.30]	–
Cable car - Bus	2	–	–	[93.74–103.09]
Cable car – BRT	2	–	–	[92.74–100.88]
Cable car – BRT – Walk	3	–	–	[98.72–106.14]
Cable car – BRT – Bus	3	–	–	[95.41–101.00]

References

Andersen, M.H.G., Johannesson, S., Fonseca, A.S., Clausen, P.A., Saber, A.T., Roursgaard, M., et al., 2019. Exposure to air pollution inside electric and diesel-powered passenger trains. Environ. Sci. Technol. 53, 4579–4587. https://doi.org/10.1021/acs.est.8b06980.

Canon Rubiano, L., Portabales Gonzalez, I., Flor, L., Duarte, D., Sierra Valdivieso, L., 2020. Urban Aerial Cable Cars as Mass Transit Systems : Case Studies, Technical Specifications, and Business Models. Lima. Available: https://documents.worldbank.org/en/publication/documents-reports/documentdetail/140251611326011396/urban-aerial-cable-cars-as-mass-transit-systems-case-studies-technical-specifications-and-business-models.

Carlet, F., 2016. An overview of aerial ropeway transit and its potential in urban environments. SBE2016 Towards Post Carbon Cities.

Cepeda, M., Schoufour, J., Freak-Poli, R., Koolhaas, C.M., Dhana, K., Bramer, W.M., et al., 2017. Levels of ambient air pollution according to mode of transport: a systematic review. Lancet Public Health https://doi.org/10.1016/S2468-2667(16)30021-4.

Chen, R., Samoli, E., Wong, C.M., Huang, W., Wang, Z., Chen, B., et al., 2012. Associations between short-term exposure to nitrogen dioxide and mortality in 17 chinese cities: the China air pollution and health effects study (CAPES). Environ. Int. https://doi.org/10.1016/j.envint.2012.04.008.

Chen, H., Goldberg, M.S., Burnett, R.T., Jerrett, M., Wheeler, A.J., Villeneuve, P.J., 2013. Long-term exposure to traffic-related air pollution and cardiovascular mortality. Epidemiology https://doi.org/10.1097/EDE.0b013e318276c005.

de Nazelle, A., Fruin, S., Westerdahl, D., Martinez, D., Ripoll, A., Kubesch, N., et al., 2012. A travel mode comparison of commuters' exposures to air pollutants in Barcelona. Atmos. Environ. https://doi.org/10.1016/j.atmosenv.2012.05.013.

Dennekamp, M., Mehenni, O.H., Cherrie, J.W., Seaton, A., 2002. Exposure to ultrafine particles and PM 2.5 in different micro-environments. Ann. Occup. Hyg. 46, 412–414. https://doi.org/10.1093/ANNHYG/46.SUPPL_1.412.

Dons, E., Int Panis, L., van Poppel, M., Theunis, J., Wets, G., 2012. Personal exposure to black carbon in transport microenvironments. Atmos. Environ. https://doi.org/10.1016/j.atmosenv.2012.03.020.

Freedson, P.S., Melanson, E., Sirard, J., 1998. Calibration of the computer science and applications. Inc. Accelerometer. Med Sci. Sports Exerc. 30, 777–781.

Gan, W.Q., Koehoorn, M., Davies, H.W., Demers, P.A., Tamburic, L., Brauer, M., 2011. Long-term exposure to traffic-related air pollution and the risk of coronary heart disease hospitalization and mortality. Environ. Health Perspect. https://doi.org/10.1289/ehp.1002511.

Global Road Safety Facility, Institute for Health Metrics and Evaluation, The World Bank, Institute for Health Metrics and Evaluation, 2014. Transport for Health:The Global Burden of Disease From Motorized Road Transport. 2014. The Word Bank, Washington, DC. https://doi.org/10.1016/B978-012373960-5.00335-X Washington D.C, WA: IHME.

Gouveia, N., Kephart, J.L., Dronova, I., McClure, L., Granados, J.T., Betancourt, R.M., et al., 2021. Ambient fine particulate matter in latin american cities: levels, population exposure, and associated urban factors. Sci. Total Environ. 772, 145035. https://doi.org/10.1016/j.scitotenv.2021.145035.

Grahame, T.J., Schlesinger, R.B., 2010. Cardiovascular health and particulate vehicular emissions: a critical evaluation of the evidence. Air Qual. Atmos. Health https://doi.org/10.1007/s11869-009-0047-x.

Gulliver, J., Briggs, D.J., 2004. Personal exposure to particulate air pollution in transport microenvironments. Atmos. Environ. https://doi.org/10.1016/j.atmosenv.2003.09.036.

Guzman, L.A., Oviedo, D., Bocarejo, J.P., 2017. City profile: the Bogotá metropolitan area that never was. Cities 60, 202–215. https://doi.org/10.1016/J.CITIES.2016.09.004.

Guzman, L.A., Arellana, J., Alvarez, V., 2020. Confronting congestion in urban areas: developing sustainable mobility plans for public and private organizations in Bogotá. Transp. Res. Part A Policy Pract. 134, 321–335. https://doi.org/10.1016/J.TRA.2020.02.019.

Guzman, L.A., Cantillo-Garcia, V.A., Arellana, J., Sarmiento, O.L., 2022. User expectations and perceptions towards new public transport infrastructure: evaluating a cable car in Bogotá. Transportation (Amst), 1–21 https://doi.org/10.1007/S11116-021-10260-X/FIGURES/4.

Health Effects Institute, 2010. Traffic-Related Air Pollution: A Critical Review of the Literature on Emissions, Exposure, and Health Effects. Boston. Available: https://www.healtheffects.org/publication/traffic-related-air-pollution-critical-review-literature-emissions-exposure-and-health.

Heinrichs, D., Goletz, M., Lenz, B., 2017. Negotiating territory: strategies of informal transport operators to access public space in urban Africa and Latin America. Transp. Res. Procedia 25, 4507–4517. https://doi.org/10.1016/J.TRPRO.2017.05.346.

Johansson, C., Burman, L., Forsberg, B., 2009. The effects of congestions tax on air quality and health. AtmEn 43, 4843–4854. https://doi.org/10.1016/J.ATMOSENV.2008.09.015.

Kaur, S., Nieuwenhuijsen, M.J., Colvile, R.N., 2007. Fine particulate matter and carbon monoxide exposure concentrations in urban street transport microenvironments. Atmos. Environ. https://doi.org/10.1016/j.atmosenv.2007.02.002.

Kawahara, J., Tanaka, S., Tanaka, C., Aoki, Y., Yonemoto, J., 2011. Estimation of daily inhalation rate in preschool children using a tri-axial accelerometer: a pilot study☆. Sci. Total Environ. 409, 3073–3077. https://doi.org/10.1016/j.scitotenv.2011.04.006.

Levy, J.I., Dumyahn, T., Spengler, J.D., 2002. Particulate matter and polycyclic aromatic hydrocarbon concentrations in indoor and outdoor microenvironments in Boston, Massachusetts. Journal of Exposure Science & Environmental Epidemiology 12 (2), 104–114. https://doi.org/10.1038/sj.jea.7500203.

Madueño, L., Kecorius, S., Löndahl, J., Müller, T., Pfeifer, S., Haudek, A., et al., 2019. A new method to measure real-world respiratory tract deposition of inhaled ambient black carbon. Environ. Pollut. 248, 295–303. https://doi.org/10.1016/J.ENVPOL.2019.02.021.

Matz, C.J., Stieb, D.M., Egyed, M., Brion, O., Johnson, M., 2018. Evaluation of daily time spent in transportation and traffic-influenced microenvironments by urban canadians. Air Qual. Atmos. Health https://doi.org/10.1007/s11869-017-0532-6.

Maynard, D., Coull, B.A., Gryparis, A., Schwartz, J., 2007. Mortality risk associated with short-term exposure to traffic particles and sulfates. Environ. Health Perspect. https://doi.org/10.1289/ehp.9537.

Morales Betancourt, R., Galvis, B., Balachandran, S., Ramos-Bonilla, J.P., Sarmiento, O.L., Gallo-Murcia, S.M., et al., 2017. Exposure to fine particulate, black carbon, and particle number concentration in transportation microenvironments. Atmos. Environ. 157, 135–145. https://doi.org/10.1016/j.atmosenv.2017.03.006.

Morales Betancourt, R., Galvis, B., Rincón-Riveros, J.M., Rincón-Caro, M.A., Rodriguez-Valencia, A., Sarmiento, O.L., 2019. Personal exposure to air pollutants in a bus rapid transit system: impact of fleet age and emission standard. Atmos. Environ. 202, 117–127. https://doi.org/10.1016/j.atmosenv.2019.01.026.

Morales Betancourt, R., Galvis, B., Mendez-Molano, D., Rincón-Riveros, J.M., Contreras, Y., Montejo, T.A., et al., 2022. Toward cleaner transport alternatives: reduction in exposure to air pollutants in a mass public transport. Environ. Sci. Technol. https://doi.org/10.1021/ACS.EST.1C07004 acs.est.1c07004.

ONU, 2017. Nueva Agenda Urbana. Habitat III. Conferencia de las Naciones Unidas sobre la Vivienda y el Desarrollo Urbano Sostenible ISBN: 978-92-1-132736-6.

Peretz, A., Sullivan, J.H., Leotta, C.A., Sands, F.N., Allen, J., Carlsten, C., et al., 2008. Diesel exhaust inhlation elicits acute vasoconstriction in vivo. AvailableEnviron. Health Perspect. 116, 937–942. https://pubmed.ncbi.nlm.nih.gov/18629317/.

R Core Team, 2019. R: A language and environment for statistical computing. Vienna, Austria, Austria: R Foundation for Statistical Computing. Available: http://www.r-project.org/index.html.

Rodriguez-Valencia, A., Rosas-Satizabal, D., Paris, D., 2019. Importance-Performance Analysis in Public Transportation: Methodological Revision for Practical Implementation. 2673, pp. 710–723. https://doi.org/10.1177/0361198118825125.

Sarmiento, O.L., Siri, J., Rodriguez, D., Higuera-Mendieta, D.D., Gonzalez, S., Montero, S., et al., 2017. Sustainable Transport and Urban Health: The Lessons of Latin America. Bogotá, pp. 1–6.

Sarmiento, O.L., Higuera-Mendieta, D., Wilches-Mogollon, M.A., Guzman, L.A., Rodríguez, D.A., Morales, R., et al., 2020. Urban transformations and health: methods for TrUST—a natural experiment evaluating the impacts of a mass transit cable car in Bogotá, Colombia. Front Public Health 8, 64. https://doi.org/10.3389/fpubh.2020.00064.

Secretaría Distrital de Movilidad, 2019. Encuesta de Movilidad 2019. Bogotá.

Targino, A.C., Krecl, P., Cipoli, Y.A., Oukawa, G.Y., Monroy, D.A., 2020. Bus commuter exposure and the impact of switching from diesel to biodiesel for routes of complex urban
